# Headgear Accessories Classification Using an Overhead Depth Sensor

**DOI:** 10.3390/s17081845

**Published:** 2017-08-10

**Authors:** Carlos A. Luna, Javier Macias-Guarasa, Cristina Losada-Gutierrez, Marta Marron-Romera, Manuel Mazo, Sara Luengo-Sanchez, Roberto Macho-Pedroso

**Affiliations:** Department of Electronics, University of Alcala, Ctra. Madrid-Barcelona, km.33,600, 28805 Alcalá de Henares, Spain; carlos.luna@uah.es (C.A.L.); javier.maciasguarasa@uah.es (J.M.-G.); marta.marron@uah.es (M.M.-R.); manuel.mazo@uah.es (M.M.); sara.luengo@depeca.uah.es (S.L.-S.); roberto.macho@depeca.uah.es (R.M.-P.)

**Keywords:** headgear accessories classification, time-of-flight sensor, feature extraction, semantic features, depth maps, overhead camera

## Abstract

In this paper, we address the generation of semantic labels describing the headgear accessories carried out by people in a scene under surveillance, only using depth information obtained from a Time-of-Flight (ToF) camera placed in an overhead position. We propose a new method for headgear accessories classification based on the design of a robust processing strategy that includes the estimation of a meaningful feature vector that provides the relevant information about the people’s head and shoulder areas. This paper includes a detailed description of the proposed algorithmic approach, and the results obtained in tests with persons with and without headgear accessories, and with different types of hats and caps. In order to evaluate the proposal, a wide experimental validation has been carried out on a fully labeled database (that has been made available to the scientific community), including a broad variety of people and headgear accessories. For the validation, three different levels of detail have been defined, considering a different number of classes: the first level only includes two classes (hat/cap, and no hat/cap), the second one considers three classes (hat, cap and no hat/cap), and the last one includes the full class set with the five classes (no hat/cap, cap, small size hat, medium size hat, and large size hat). The achieved performance is satisfactory in every case: the average classification rates for the first level reaches 95.25%, for the second one is 92.34%, and for the full class set equals 84.60%. In addition, the online stage processing time is 5.75 ms per frame in a standard PC, thus allowing for real-time operation.

## 1. Introduction

People detection and human behavior analysis in video-surveillance applications are already classic research topics in computer vision, artificial intelligence, and machine learning areas but are still an open issue, with some commercial solutions performing some basic tasks with reasonable accuracy but still not available for other higher-level understanding tasks. One of the main reasons for this is the context diversity of the in-the-wild real video-surveillance images. Nevertheless, technological evolution as well as machine learning algorithmic contributions facilitate and speed up more and more reliable and robust context aware solutions, making them in order to contribute to solving this part of the problem.

In addition, identity preservation is the other open issue that should be considered in order to obtain the commercial solutions demanded by the everyday most connected and technologically informed world. Time-of-Flight (ToF) cameras, that obtain depth images from the surveilled scenarios, give high-resolution images suitable for the image analysis tasks needed by the surveillance application pursuit, while at the same time preserving the privacy of surveilled people.

Within this context, this work presents a proposal for only using depth information that is acquired using an overhead ToF camera [1,2], to be applied to the classification of different headgear accessories (Throughout the paper, we will use “headgear accessories”, “head accessories” or simply “accessories” to refer to different types of hats or caps). The camera placement was selected in zenithal position, as it greatly avoids occlusions (thus facilitating the task of people detection and segmentation), the camera installation is easy, and the image acquisition position contributes to preserving the privacy of the persons in the scene. 

The selected accessories are limited by the camera position, which only provides a top-view. Because of this, the proposal allows for detecting and classifying different headgear accessories such as hats, caps, etc. when worn by people in the overhead scene under surveillance. It is worth highlighting the fact that this information is enough for obtaining semantic features of the upper-body of people in the scene, which is useful in the surveillance applications focused on by the scene understanding commercial solution pursuits. 

In fact, the interest in classifying people under surveillance by their headgear accessories (caps, hats, etc.), and preserving their identity, is justified as it provides additional semantic information that can be used by higher level processing systems (such as tracking, re-identification, scene understanding, etc.), where a combination of different semantic features is normally used.

Due to this, the classification of human accessories in semantic video analysis tasks has attracted a great interest in recent years within the research community [3], since it allows for semantically describing people for different applications, such as security and biometrics [4,5], surveillance [6], human computer interaction [7], or oriented marketing [8]. Recently, there has been an increasing number of approaches proposed in the scientific literature, which are able to extract human attributes such as gender, ethnicity, age, hairstyle or clothing, focused on in such applications. 

The early approaches [8,9,10] in these areas used RGB (Red, Green, Blue) data for extracting such semantic features. For example, the authors of [9] proposed a method for recognizing attributes, such as gender, hair style and different types of clothes (hats, t-shirts, shorts, jeans, long hair, glasses, long sleeves, long pants), in people under variations in viewpoint and pose, based on attribute classifiers and a discriminative model. In [10], the authors proposed a novel hair style retrieval algorithm from face images. The proposal in [8] generates a list of semantic accessories for clothes worn by people in RGB images, using attribute classifiers and Conditional Random Fields. In that proposal, some variations in the viewpoint are allowed, but in most of the images, people are facing the camera. In our work, we contribute to this area by addressing the problem of attribute classification (headgear accessories) using depth data from an overhead ToF camera. 

In recent years, most of the approaches have started using RGB-D data, which are less sensitive to indoor ambient conditions, providing depth information in addition to RGB. The proposal in [6] detects fine-grained human attributes (gender, wearing a backpack, talking on a cell phone, wearing glasses, short sleeves, long pants) in surveillance environments. The proposal in [11] allows detecting gender, as well as other attributes such as hair style or clothing [7] in RGB-D images, not only from frontal body views, but also from side and back views. However, none of the previous works use depth information only or top-view images, and thus do not take into account the privacy issues so sensible in surveillance applications. On the other hand, the work in [12] proposes an approach for human attribute analysis, such as gender and whether they have bags with them, based on multi-layer classification, and using top-view RGB camera images. In our case, we still want to avoid the use of RGB data for privacy consideration issues.

There are other works in the literature that use overhead depth cameras [13,14,15,16,17,18,19,20] in surveillance applications. The use of depth cameras allows for working in changing light conditions [13], as well as preserving the users’ privacy [15]. At the same time, the use of an overhead camera reduces the occlusions [14,17,18,19,20] and the variation of target features [16]. 

All of the cited works are focused on detecting and tracking or counting people, not allowing the obtaining of any semantic attribute from them. Specifically, the authors of [13] propose the use of a ToF depth sensor to allow people tracking, but it only works if people enter the scene well separated, in order to avoid occlusions. The work presented in [14] obtains better results for multiple people, using an approach based on a normalized Mexican Hat Wavelet, but it also fails if people are close to each other (as we showed in [21]). In the proposal in [15], after removing the noise and background, any group of connected depth measurements (blobs) whose height is over 90 cm is detected as a person, thus allowing the easy generation of false positives as the detector criteria is only based on height. Other works, such as Refs. [16,19], use RGB images for people counting. These works only include a detector, but not a classification stage, so that they cannot discriminate between people and other objects in the scene. On the other hand, the proposals described in [17,18,20] include a classification stage that allows for reducing the number of false positives. More specifically, the works described in [17,20] are based on the human head and shoulders’ physical structure for obtaining a person overhead descriptor. These approaches are able to discriminate between people and other elements in the scene, but their detection rates decrease if people are close to each other. Moreover, they suffer from a lack of robustness if people wear complements such as hats, caps, backpacks, etc.

The proposal we describe in this paper not only allows for detecting people from an overhead ToF camera, but is also able to handle scenes with people that are close to each other (being based on our previous proposal [21]), and to discriminate between people wearing different accessories that can have a variety of appearances, such as hats and caps. 

The structure of the paper is as follows: Section 2 describes the accessories classification algorithmic proposal; Section 3 covers the experimental work, including the description of the experimental setup, the performance metrics used and the obtained results, both in terms of classification rates and computational cost; and Section 4 draws the main conclusions of the paper. 

## 2. Accessories Classification Algorithm 

As described in the introduction, in this work, we propose an approach for the classification of headgear accessories only using depth information provided by a ToF sensor located in an overhead position. The camera position, which only provides a top-view, allows for preserving the privacy of the users and restricts the type of accessories that can be detected and classified. Our proposal focuses on the design of a robust processing strategy, including the estimation of a meaningful feature vector containing the relevant information that could be extracted from an overhead ToF camera, and its application on the headgear accessories classification. Within this context, the proposed method is based on Principal Components Analysis (PCA), although any classification method could be applied. The classification procedure uses an eight-component feature vector that is related to the geometry of the surface information of the people head and shoulders, designed ad hoc for this application.

**Figure 1:**
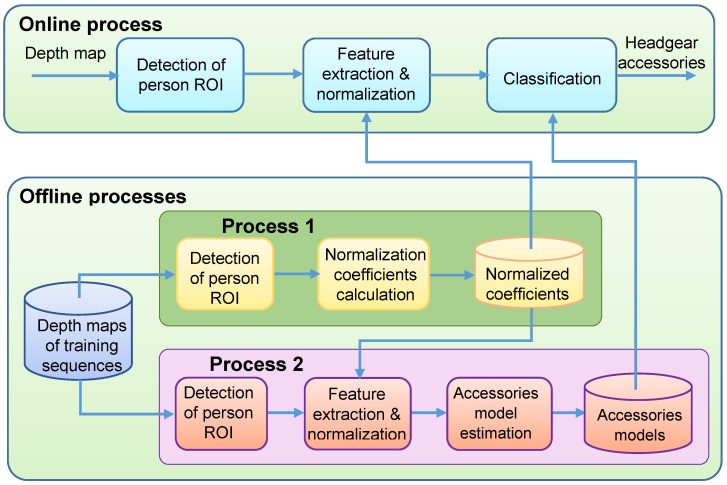
General block diagram of the proposed method.

A general block diagram of the proposed method is shown in Figure 1. There are three main processes, two offline processes and an online one. In the offline Process 1, some coefficients that are used to normalize the feature vector are estimated. In the offline Process 2, the model estimation is carried out. In our case, five different models are generated: one for people without any accessories, another one for people wearing caps, as well as three more models for people wearing different types (sizes) of hats. In the online process, the recovery error of the feature vector for each model is calculated, and the model with the smallest error is assumed to correspond to the accessory class that the users actually wear. 

In the next sections, we describe in detail each stage of the online and offline processes shown in Figure 1. It is worth mentioning that some of the stages, such as the preprocessing of the depth maps or the detection of the person ROIs (Regions of Interest), are common to both (online and offline) processes. 

### 2.1. Preprocessing of Depth Maps

The depth maps are captured using a ToF camera fixed to the ceiling at height $h_{c}$ from the floor, the camera optical axis being perpendicular to the floor plane. Some of the problems exhibited in depth maps captured by ToF cameras are the great number of invalid pixels that appear in the object borders, the motion artifacts, and the high noise level [22,23,24]. In this work, the invalid pixels are corrected by means of the interpolation of the nearest valid neighboring pixels, using the algorithm proposed in [21]. Moreover, in order to reduce the noise in the depth information, a nine-element mean filter is applied. Next, the depth information is translated to the floor origin, obtaining information about the actual height of each pixel in the scene. The height matrix obtained after the preprocessing is denoted by **H**. 

### 2.2. Detection of a Person ROI

In this work, the body elements of interest are the head and shoulders areas, so that it is necessary to determine which pixels belong to these body parts. To achieve this objective, a region of interest (ROI) is defined as the set of pixels that belong to a person while also having height values within a height range that corresponds to the areas of interest. To determine a person’s ROI, the contour detection algorithm to be used has to take into account the sparsity of the depth data, and the conditions imposed by the overhead camera position [21,25]. After that, it is necessary to find the maximum height value ($h_{max}$) within that contour and, finally, select the pixels whose heights are between $h_{max}$ and the minimum height of interest $h_{min}$. Based on anthropometricconsiderations [26,27], we assume that: $h_{min}=h_{max}-40\;\mathrm{cm}$. In what follows, the set of selected pixels will be represented by θ(h).

### 2.3. Feature Extration and Normalization

In [21], we used a feature vector that described the pixel density associated to the head and shoulders surface within the corresponding ROI. In that work, we used a feature vector composed of six components: five of them related to the visible people surfaces at different heights, and the sixth component corresponding to the eccentricity of the person head. These six components are valid to describe the upper part of a person and to detect if a particular object in the scene corresponds to a person or not, but it is not precise enough to be used in the classification of headgear accessories (as they focus on only modeling the upper part of the head and the shoulders, so not being aimed at the intermediate region in which the headgear accessories key elements will be located). 

**Figure 2:**
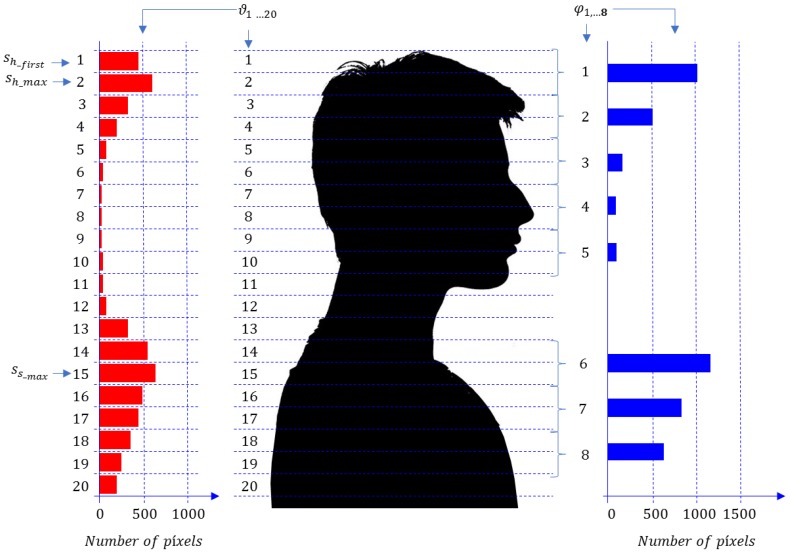
Example of slice segmentation for a person. The number of pixels (depth measurements) in each slice is shown on the left (ϑ). The values of the feature vector components ($\varphi =\left[\varphi_{1},\;\varphi_{2},\ldots ,\varphi_{8}\right]$) are shown on the right.

To increase the system robustness in the accessories classification task, we propose a new feature vector that is composed of eight components, which describe the geometrical structure of the head and shoulders areas, and in a more detailed way as compared with [23]: five of them describe the head area geometry, and the other three describe the shoulders geometry. To show an example of the geometrical regions for which the feature vector is providing information, and to allow a proper understanding of how it works, we have generated Figure 2. In this Figure, a side-on portrait view of a person is shown, indicating the division in horizontal slides from which the calculations are being carried out, applying the following procedure:

1. Calculate the histogram of pixels (depth measurements) along the heights of interest θ(h) (between $h_{max}$ and $h_{min}$), with a high enough number of intervals. In this work, 20 intervals were used, as proposed in [21], taking into account geometrical considerations (Figure 2 shows this division in 20 slices). That is, we count the number of pixels in 20 slices of 2 cm of height, obtaining a vector $\vartheta =\left[\vartheta_{1}\;\ldots \vartheta_{s}\;\ldots \vartheta_{20}\right]$, where $\vartheta_{s}$ is the number of pixels in section *s* (being *s* = 1, 2, …, 20). The highest section of a given person corresponds to *s* = 1, as shown in Figure 2.
2. Estimate the first top section of the head, $s_{h\_first}$. To do this, we first find the section of the head with the maximum number of pixels in the first three elements of ϑ: $s_{h\_max}=arg\max\left(\vartheta_{1}\;,\vartheta_{2}\;,\vartheta_{3}\right)$. Then, we assume that:
  (1) $$\begin{array}{c} If\;s_{h\_max}=1:\;s_{h{\_}_{first}}=s_{h\_max}\\ If\;s_{h\_max}>1\;and\;NT\cdot \vartheta_{s_{h{\_}_{max}}-1\;}\geq \vartheta_{s_{h\_max}\;}:\;s_{h{\_}_{first}}=s_{h\_max}-1\\ If\;s_{h\_max}>1\;and\;NT\cdot \vartheta_{s_{h{\_}_{max}}-1\;}<\vartheta_{s_{h\_max}\;}:\;s_{h{\_}_{first}}=s_{h\_max} \end{array}\},$$
  where NT is the number of times that the numbers of pixel in section $\vartheta_{s_{h_{\_max}}-1}$ must be higher than the number of pixels in section $\vartheta_{s_{h\_max}}$ to assume that the top section is $\vartheta_{s_{h{\_}_{max}}-1}$. This step is necessary to reduce the error in the person height estimation, due to the noise in the depth maps, and for the use of small accessories or specific hair configurations (such as tied in a tail). The value of *NT* has been obtained empirically, and it was determined that, with values of NT between 10 and 20, similar results were obtained. The estimated value of the person height without noise, $h_{p}$, will be obtained as:
  (2) $$h_{p}=h_{max}-2\;\mathrm{cm}\cdot \left(s_{h{\_}_{first}}-1\right).$$
3. Find the five components of the feature vector (φ) that describe the head structure. Each component is composed of two consecutive elements of ϑ organized as follows:
  (3) $$\varphi_{i}=\vartheta_{2i-2+s_{h{\_}_{first}}}\;+\vartheta_{2i-1+s_{h{\_}_{first}}},\;\mathrm{for}\;i=1,\;2,\ldots ,\;5.$$
4. Estimate the maximum section of the shoulders, $s_{s\_max}$. Taking into account the human morphology, we assume that $s_{s\_max}$ can be found starting at 22 cm from the top of the head, i.e.:
  (4) $$s_{s{\_}_{max}}=arg\max\left(\vartheta_{11+s_{h{\_}_{first}}},\ldots ,\vartheta_{16}\right).$$
5. Find the three components of the feature vector (φ) that describe the shoulders’ structure. Each component is composed of two consecutive elements of ϑ organized as follows:
  (5) $$\varphi_{i+5}=\vartheta_{2\left(i-1\right)-1+s_{s{\_}_{max}}}\;+\vartheta_{2\left(i-1\right)+s_{s{\_}_{max}}},\;\mathrm{for}\;i=1,\;2,\;3.$$
  Each component $\varphi_{i}$ corresponds to the number of pixels of a section of 4 cm, so that it depends on the person’s height, thus it is necessary to normalize them.
6. Determine the normalized feature vector (Ψ) that will be obtained dividing the components of the feature vector (φ), by the estimated value of its first component ($\hat{\varphi_{1}}$):
  (6) Ψ=φφ1^.

The next section describes how to estimate the value of the normalization parameter $\hat{\varphi_{1}}$.

### 2.4. Calculation of Normalization Coefficients

To make φ independent of a person’s height ($h_{p}$), the relationship between $h_{p}$ and $\varphi_{1}$ must be determined. For a training sample composed of a set of people with heights between 140 cm and 213 cm, the values of $h_{p}$ and $\varphi_{1}$ were calculated (using the steps 1, 2 and 3 described in Section 2.3). From the analysis of the results, we observed a quadratic relationship between them, so that we assumed that:

(7) $$\hat{\varphi_{1}}=a_{0}+a_{1}\cdot h_{p}+a_{2}\cdot h_{p}^{2},$$

where $a_{0}$, $a_{1}$ and $a_{2}$ are the coefficients that must be estimated.

Using a nonlinear least squares approximation method, the obtained values were: $a_{0}=1998.0$, $a_{1}=-24.62$ and $a_{2}=0.092$, which empirically showed to be adequate for the normalization task. 

### 2.5. Attribute Model Estimation

**Figure 3:**
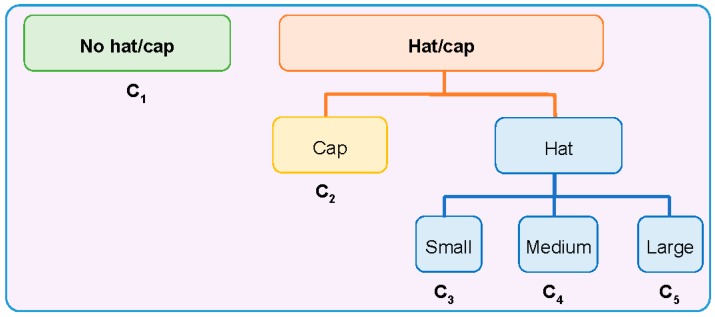
Diagram of the class organization in three different levels of detail.

In this work, we define five classes (C_1_, C_2_, … , C_5_) in the classification of headgear accessories (see Figure 3). With the objective of simplifying the notation and following the same order in which they appeared previously, these classes will be referred to as α=1,…, 5 (being α=1 for C_1_ … α=5 for C_5_).

We initially selected a PCA classification strategy for its simplicity and low computational requirements. PCA is a well-known method that has been widely used for representing high-dimensional data in a low-dimensional space [28,29], and also for classification [21,30,31]. However, any other classification method, such as a Multi-Layer Perceptrons (MLPs), Support Vector Machines (SVMs), etc., could have been used, provided that the feature vector contains meaningful information for the target task.

The PCA classifier requires an offline training process to estimate a model for each class α. Therefore, each of the models is composed of an average vector ${\bar{\Psi }}_{\alpha }$, a scatter matrix $S_{\alpha }$, and a transformation matrix $U_{\alpha }$:

(8) $${\bar{\Psi }}_{\alpha }=\frac{1}{N_{\alpha }}\sum_{i=1}^{N_{\alpha }}\Psi_{\alpha i},$$

(9) $$S_{\alpha }=\sum_{i=1}^{N_{\alpha }}(\Psi_{\alpha i}-{\bar{\Psi }}_{\alpha }){\left(\Psi_{\alpha i}-{\bar{\Psi }}_{\alpha }\right)}^{T},$$

where $N_{\alpha }$ is the number of training vectors that represents the class α. 

The matrices $U_{\alpha }$ are formed by the eigenvectors associated with the largest (principal) eigenvalues of the scatter matrices $S_{\alpha }$. Following the criterion that the average normalized residual quadratic error (RMSE) is higher than 90%, three eigenvectors were finally considered in this work.

### 2.6. Classification 

The classification of each vector Ψ corresponding to a person in the scene is described in Algorithm 1, where the feature vector Ψ of a person is projected on the transformed space and recovered to the original space for each class. A person is classified as corresponding to the class α when the Mahalanobis distance between $\Phi_{\alpha }$ and ${\hat{\Phi }}_{\alpha }$ is the smallest one among all of the considered classes.
**Algorithm 1** Classification1:**Input:** feature vector Ψ, average vector ${\bar{\Psi }}_{\alpha }$, scatter matrix $S_{\alpha }$, transformation matrix $U_{\alpha }$, number of classes =5.2:**For**
α=1 to 5
**do**
3: $\Phi_{\alpha }=\Psi -\;{\bar{\Psi }}_{\alpha }$ // *Difference between the feature vector and the average vector* //4: $\Omega =U_{\alpha }^{T}\Phi_{\alpha }$ // *Projection of*
$\Phi_{\alpha }$
*in the transformed space*//5: ${\hat{\Phi }}_{\alpha }=U_{\alpha }\Omega$ // *Recovery of*
$\Phi_{\alpha }$
*in the original space* //6: $\Delta \Phi_{\alpha }=\Phi_{\alpha }-\;{\hat{\Phi }}_{\alpha }$ // *Difference between the vector*
$\Phi_{\alpha }$
*and its recovery*
${\hat{\Phi }}_{\alpha }$ //7: $\varepsilon_{\alpha }=\Delta \Phi_{\alpha }^{T}\cdot S_{\alpha }^{-1}\cdot \Delta \Phi_{\alpha }$ // *Recovery error*
$\varepsilon_{\alpha }$
*= Mahalanobis distance between*
$\Phi_{\alpha }$
*and*
${\hat{\Phi }}_{\alpha }$//8: **If**
$\varepsilon_{\alpha }<\varepsilon_{min}$
**then**9:  $\varepsilon_{min}=\varepsilon_{\alpha }$
**and**
Class= α // *Class*
α
*has now the smallest recovery error* //10: **End If**11:**End For**12:**Output:**
Class //*Class with the smallest recovery error*//

## 3. Experimental Work

### 3.1. Experimental Setup

**Figure 4:**
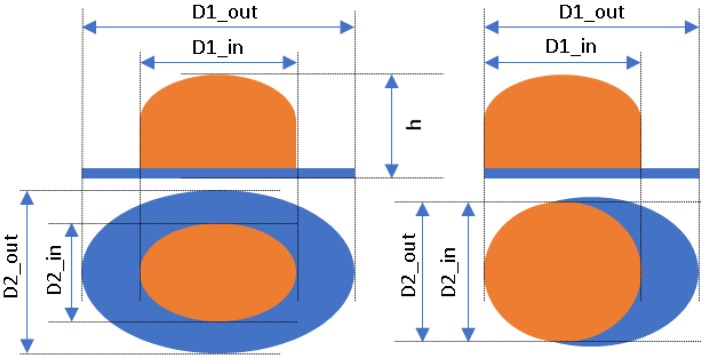
Diagram of the sizes of the accessories used in Table 1, for hats (**left**) and caps (**right**).

In order to provide data for training and evaluating the proposal, we have used part of the GOTPD2 database (available at [32]). GOTPD2 was recorded in an indoor environment using two Kinect v2 devices (Microsoft, Redmon, WA, USA) located at a height of 3.4 m, perpendicularly pointing towards the room floor. The recordings tried to cover a broad variety of conditions, with scenarios comprising:

- Single people scenarios, as the target is the classification of head style/accessories configuration, with a total of 25 different persons involved.
- People without accessories (no hat nor cap) and different hairstyle configurations (short, long and with/without ponytail).
- People with 21 different headgear accessories, 17 hats (of different sizes) and four caps. Table 1 shows the details of the different accessories used, including information on their size (with the naming conventions indicated in Figure 4).

The dataset was divided into two subsets, one for training and the other one for testing. The subsets are fully independent, so that no person nor accessory present in the training subset was present in the testing subset. The columns Training Accessories and Testing Accessories in Table 1 show the accessories used for training and testing, respectively. It can be seen that just one hat/cap per class was used for training, and a variety of them was used for testing. Preliminary experiments were carried out to decide on the amount of training material required, and using a single hat/cap per class for training proved to be good enough, allowing us to use a bigger testing database to increase the statistical significance of the results. 

**Figure 5:**
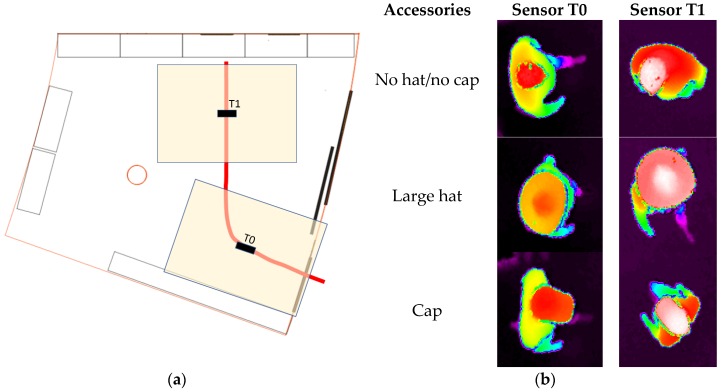
(**a**) top view of the room in which the recordings took place; (**b**) example images acquired by T0 and T1 depth sensors, belonging to different classes.

The database contains sequences in which the users were instructed on how to move under the camera in linear directions (parallel or diagonal to the captured area, to allow for proper coverage of the recording area). A top-view of the room, in which the recording took place, and some example images belonging to different classes are shown in Figure 5. In Figure 5a, T0 and T1 are the two overhead ToF cameras used to record the sequences, the color-filled rectangles show the camera field of view at the floor level, and the thick line shows the trajectory followed by the users in the recordings. In Figure 5b, the height values are shown using a color scale representation.

We run preliminary experiments to assess the actual precision of the Kinect devices when estimating the depth values. We found that errors in height estimation were below 1 cm for users in static position, which is consistent with previous reports in the literature (such as [33], where the authors report a maximum error of 4 cm at the maximum range of the sensor, but our measured elements are much nearer the sensor, as we evaluate people standing).

Table 2 and Table 3 show the details of the training and testing subsets, respectively. #Sequences refers to the number of recorded sequences, #Frames refers to the number of all of the (useful) frames with people present. In Table 3, the number of sequences and frames is shown as T0 + T1, as they refer to the recordings done with each of the two overhead cameras: T0 and T1.

The Level 1 grouping just identifies the three main *broad* classes, namely Caps, Hats, and none of them (referred to as No hat/cap). In the Level 2 grouping, the classification was further refined for the Hats class, considering their size: large, medium and small. This refinement is somehow fuzzy, as the frontiers between each of these classes is somehow arbitrary, but we want to stress the fact that we are interested in providing additional semantic labels, with reasonable performance, to characterize people in the scene. In our case, we did this grouping mainly considering the external diameters of each hat (see size information in Table 1, considering the size definitions of Figure 4).

### 3.2. Performance Metrics

To evaluate the performance of our proposal, we will provide the full confusion matrices for the experiments carried out, and the average classification rates. In the case of the confusion matrices, we will also include the confidence interval values for a confidence level of 95%.

### 3.3. Results and Discussion

Several experiments were run in order to assess the capabilities of the proposed system in coping with different requirements, namely as above:

- Level 2 classification grouping: in which five classes were considered, i.e., large size hat, medium size hat, small size hat, cap and no hat/cap.
- Level 1 classification grouping: in which three classes were considered, i.e., hat, cap and no hat/cap.
- Accessory–no accessory classification: in which two classes were considered, i.e., hat/cap, and no hat/cap.

Table 4 shows the results for the level 2 classification task. The average classification rate is 84.60%, which may seem a bit low to be usable in a real context, but we have to take into account again that the frontier between hat sizes is somehow arbitrary. This can be clearly seen in the grayed areas that show, for every class, the somehow high confusion between adjacent hat sizes, and between using a cap and wearing nothing on the head (actually, a cap adjusts to the general head shape, except for its frontal shelter/cover).

Table 5 shows the results for the level 1 classification task. The average classification rate in this case is 92.33%. Again, a non-negligible confusion between the cap class and the no hat/cap class can be noticed, for the same reason stated above.

Table 6 shows the results for the hat/cap–no hat/cap classification task. The average classification rate is 95.25%. From Table 6, it is also easy to find out the sensitivity (true positive rate), which equals 95.22%, the specificity (true negative rate), which equals 95.61%, and the F1 score, which equals 95.41%.

As a final comment, even if the classification performance may seem relatively low for the level 2 classification grouping, we want to stress that the proposal objective is providing semantic labels to be used by higher level processing systems. In this scenario, even if the classification is not perfect, it can provide additional information that will be useful in the higher levels. 

### 3.4. Computational Cost

All of the experiments reported were run on a PC, with an Intel Quad Q9550 2.83 GHz processor (Santa Clara, CA, USA), and 4 GB RAM. The algorithmic proposal has been implemented in C under the LabWindows™/CVI (C for Virtual Instrumentation) Integrated Development Environment, and runs on a Windows 10 operating system (Microsoft, Redmon, WA, USA). The pre-process of the depth maps is the stage that presents the higher computational cost, with an average of 3.75 ms per frame. The rest of processes have a duration of 2 ms per frame, so that the overall processing time per frame is 5.75 ms, thus allowing for real-time operation of the proposal. 

Alternative classification strategies could have been applied, but we focused our efforts on the design of a meaningful feature vector containing the relevant information that could be extracted from an overhead ToF camera. The advantage of PCA is its relative simplicity, which would allow its implementation in low power computing architectures. To provide an example, in [31], we compared the PCA based proposal with another based in SVM, for a people detection task also using depth information from an overhead ToF camera. The SVM classifier outperformed the PCA based one, but the later one was 4.5 times faster. The computational simplicity of our proposal would even allow its implementation in low cost platforms such as the Raspberry Pi, which has proved to be able to solve even more complex preprocessing and classification problems (such as the one described in [34], aimed at face detection, also using a PCA based solution).

## 4. Conclusions

In this work, we have presented a procedure for real-time classification (processing time is 5.75 ms per frame) of different headgear accessories (hats, caps, no hats/caps), by only using the depth information provided by a ToF camera placed in an overhead position. The use of only depth information, compared with the use of RGB, allows for guaranteeing people’s privacy and decreasing the processing time.

Since a dataset fulfilling the requirements of the target application (depth maps acquired with an overhead ToF camera) was not available within the scientific community, a specific dataset has been recorded and made publicly available. This dataset contains depth sequences of 25 different people with short/long hairstyles without accessories (no hat nor cap), and with 21 different accessories (17 different hats and four different caps). 

A full description of our algorithm for accessories classification has been provided, including depth data preprocessing, person ROI estimation, feature extraction and normalization, and the final classification stage. The classification process has been carried out using PCA techniques. Within this context, the recorded dataset was divided into fully separated training and testing subsets that have been used to train and evaluate the proposal for different classification tasks (from two to five different classes). The results obtained are satisfactory, achieving an average classification rate of around 92% for the three-class problem (hat, cap, no hat/cap). This average improves when the number of classes is reduced, reaching 95% in the case of classifying people with and without accessories. The performance for the five classes classification task is around 85%, which is mainly due to the fuzzy frontiers between the hat classes considering different sizes. Nevertheless, the results are very good considering the target application of providing additional attributes in the field of semantic surveillance video analysis.

Future work will be focused on further exploiting the information provided by the system, generating additional quantitative features (for example, related to the actual area of the detected accessories), evaluating alternative classification strategies, and also integrating this proposal in a more general semantic video analysis tool, combined with other semantic attribute estimators, specifically oriented to user reidentification in wider areas.

## Figures and Tables

**Table 1:** Information on the head accessories (hats/caps) used.

Class	Training Accessories	Testing Accessories			
Caps	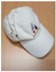 19 × 18, 27 × 18 ^2^	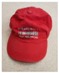 18 × 15, 25 × 15	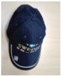 18 × 18, 27 × 18	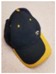 20 × 17, 27 × 17	
Large size hats ^1^	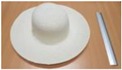 16 × 16, 38 × 38, 11	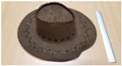 18 × 13, 38 × 35, 12	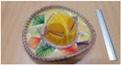 14 × 14, 34 × 34, 13	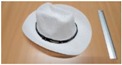 18 × 13, 36 × 31,14	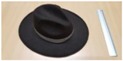 18 × 13, 35 × 32, 13
Medium size hats	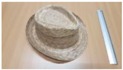 18 × 13, 29 × 26, 12	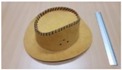 18 × 12, 32 × 26, 12	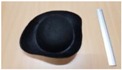 13 × 13, 24 × 31, 9	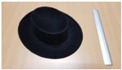 17 × 13, 32 × 27, 8	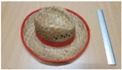 18 × 13, 32 × 30, 11
Small size hats	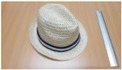 17 × 12, 27 × 23, 11	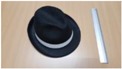 17 × 13, 26 × 24, 13	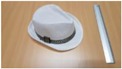 16 × 11, 15 × 20, 10	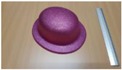 16 × 14, 26 × 23, 9	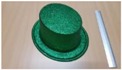 17 × 14, 28 × 24, 13
		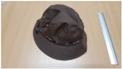 18 × 15, 28 × 25, 10	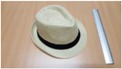 18 × 13, 27 × 25, 14		

^1^ The ruler shown is 30 cm long; ^2^ Sizes are given in cm as D1_in × D2_in, D1_out × D2_out and h, according to Figure 4.

**Table 2:** Training subset details.

#Sequences	#Frames	Level 2 Grouping	Level 1 Grouping	#Grouping1 Frames
14	492	Short hair	no hat/cap	684
6	192	Long hair		
6	399	caps	cap	399
3	384	Large size hat	hat	1064
6	289	Medium size hat		
3	391	Small size hat		
38	2147			2147

**Table 3:** Testing subset details ^1^.

#Sequences (T0 + T1)	#Frames (T0 + T1)	Level 2 Grouping	Level 1 Grouping	#Group1 Frames (T0 + T1)
17 + 16 = 33	678 + 460 = 1138	no hat/cap	no hat/cap	678 + 460 = 1138
34 + 37 = 71	1369 + 752 = 2121	cap	cap	1369 + 752 = 2121
31 + 37 = 68	1205 + 1320 = 2525	Large size hat	hats	4442 + 4708 = 9150
48 + 38 = 96	2563 + 1829 = 4392	Medium size hat		
34 + 49 = 83	674 + 1559 = 2233	Small size hat		
164 + 177 = 351	6489 + 5920 = 12,409			6489 + 5920 = 12,409

^1^ No cap/hat/user from the training subset were used here.

**Table 4:** Confusion matrix results for the level 2 classification grouping.

Type	Large Hat		Medium Hat		Small Hat		Cap		No Hat/Cap	
large hat	93.74%	±0.94%	6.14%	±0.94%	0.12%	±0.13%	0.00%	±0.00%	0.00%	±0.00%
medium hat	14.73%	±1.05%	**69.49%**	**±1.36%**	12.68%	±0.98%	0.00%	±0.00%	3.10%	±0.51%
small hat	0.00%	±0.00%	0.63%	±0.33%	**82.98%**	**±1.56%**	9.85%	±1.24%	6.54%	±1.03%
cap	0.00%	±0.00%	0.00%	±0.00%	6.69%	±1.06%	**81.19%**	**±1.66%**	12.12%	±1.39%
no hat/cap	0.00%	0.00%	0.00%	±0.00%	0.09%	±0.17%	4.31%	±1.18%	**95.61%**	**±1.19%**

**Table 5:** Confusion matrix results for the level 1 classification grouping.

Type	#Frames	Hat		Cap		No Hat/Cap	
hat	9150	94.51%	±0.47%	2.40%	±0.31%	3.08%	±0.35%
cap	2121	6.69%	±1.06%	81.19%	±1.66%	12.12%	±1.39%
no hat/cap	1138	0.09%	±0.17%	4.31%	±1.18%	95.61%	±1.19%
Frames	12,409						

**Table 6:** Confusion matrix results for the hat/cap–no hat/cap classification task.

Type	#Frames	Hat/Cap		No Hat/Cap	
hat/cap	11,271	95.22%	±0.39%	4.78%	±0.39%
no hat/cap	1138	4.39%	±1.19%	95.61%	±1.19%
#Frames	12,409				

## References

[1] Lange R., Seitz P. (2001). Solid-state Time-of-Flight range camera. IEEE J. Quantum Electron..

[2] Sell J., O’Connor P. (2014). The Xbox one system on a chip and kinect sensor. IEEE Micro.

[3] Kerker D., Jenkins M.P., Gross G.A., Bisantz A.M., Nagi R. Visual estimation of human attributes: An empirical study of context-dependent human observation capabilities. Proceedings of the 2014 IEEE International Inter-Disciplinary Conference on Cognitive Methods in Situation Awareness and Decision Support (CogSIMA).

[4] Saranya M., Cyril G.L.I., Santhosh R.R. An approach towards ear feature extraction for human identification. Proceedings of the 2016 International Conference on Electrical, Electronics, and Optimization Techniques (ICEEOT).

[5] Kim S.T., Kim D.H., Ro Y.M. Spatio-temporal representation for face authentication by using multi-task learning with human attributes. Proceedings of the 2016 IEEE International Conference on Image Processing (ICIP).

[6] Wang H.-J., Lin Y.-L., Huang C.-Y., Hou Y.-L., Hsu W. Full body human attribute detection in indoor surveillance environment using color-depth information. Proceedings of the 2013 10th IEEE International Conference on Advanced Video and Signal Based Surveillance.

[7] Linder T., Arras K.O. Real-time full-body human attribute classification in RGB-D using a tessellation boosting approach. Proceedings of the 2015 IEEE/RSJ International Conference on Intelligent Robots and Systems (IROS).

[8] Chen H., Gallagher A., Girod B. Describing clothing by semantic attributes. Proceedings of the European Conference on Computer Vision.

[9] Bourdev L., Maji S., Malik J. Describing people: A poselet-based approach to attribute classification. Proceedings of the 2011 International Conference on Computer Vision.

[10] Wang N., Ai H. Hair style retrieval by semantic mapping on informative patches. Proceedings of the First Asian Conference on Pattern Recognition.

[11] Linder T., Wehner S., Arras K.O. Real-time full-body human gender recognition in (RGB)-D data. Proceedings of the 2015 IEEE International Conference on Robotics and Automation (ICRA).

[12] Yamasaki T., Matsunami T., Chen T. (2013). Human attribute analysis using a top-view camera based on two-stage classification. IEICE Trans. Inf. Syst..

[13] Bevilacqua A., Di Stefano L., Azzari P. People tracking using a time-of-flight depth sensor. Proceedings of the 2006 IEEE International Conference on Video and Signal Based Surveillance.

[14] Stahlschmidt C., Gavriilidis A., Velten J., Kummert A., Dziech A., Czyazwski A. (2013). People detection and tracking from a top-view position using a Time-of-Flight camera. Communications in Computer and Information Science, Proceedings of the International Conference on Multimedia Communications, Services and Security, Kraków, Poland, 6–7 June 2013.

[15] Jia L., Radke R.J. (2014). Using Time-of-Flight measurements for privacy-preserving tracking in a smart room. IEEE Trans. Ind. Inform..

[16] Cai Z., Yu Z.L., Liu H., Zhang K. Counting people in crowded scenes by video analyzing. Proceedings of the 2014 9th IEEE Conference on Industrial Electronics and Applications.

[17] Galčík F., Gargalík R. Real-time depth map based people counting. Proceedings of the International Conference on Advanced Concepts for Intelligent Vision Systems.

[18] Rauter M. Reliable human detection and tracking in top-view depth images. Proceedings of the 2013 IEEE Conference on Computer Vision and Pattern Recognition Workshops.

[19] Del Pizzo L., Foggia P., Greco A., Percannella G., Vento M. (2016). Counting people by RGB or depth overhead cameras. Pattern Recognit. Lett..

[20] Vera P., Monjaraz S., Salas J. (2016). Counting pedestrians with a zenithal arrangement of depth cameras. Mach. Vis. Appl..

[21] Luna C.A., Losada C., Fuentes-Jimenez D., Fernandez-Rincon A., Mazo M., Macias-Guarasa J. (2016). Robust people detection using depth information from an overhead Time-of-Flight camera. Expert Syst. Appl..

[22] Jimenez D., Pizarro D., Mazo M., Palazuelos S. Modelling and correction of multipath interference in Time of Flight cameras. Proceedings of the 2012 IEEE Conference on Computer Vision and Pattern Recognition.

[23] Jimenez D., Pizarro D., Mazo M. (2014). Single frame correction of motion artifacts in PMD-based Time of Flight cameras. Image Vis. Comput..

[24] He Y., Liang B., Zou Y., He J., Yang J. (2017). Depth errors analysis and correction for Time-of-Flight (ToF) cameras. Sensors.

[25] Zhu L., Wong K.-H. Human tracking and counting using the kinect range sensor based on adaboost and kalman filter. Proceedings of the International Symposium on Visual Computing.

[26] Matzner S., Heredia-Langner A., Amidan B., Boettcher E.J., Lochtefeld D., Webb T. Standoff human identification using body shape. Proceedings of the 2015 IEEE International Symposium on Technologies for Homeland Security (HST).

[27] Bushby K.M., Cole T., Matthews J.N., Goodship J.A. (1992). Centiles for adult head circumference. Arch. Dis. Child..

[28] Seidenari L., Varano V., Berretti S., Del Bimbo A., Pala P. Recognizing actions from depth cameras as weakly aligned multi-part bag-of-poses. Proceedings of the 2013 IEEE Conference on Computer Vision and Pattern Recognition Workshops.

[29] Wang L., Tan T., Ning H., Hu W. (2003). Silhouette analysis-based gait recognition for human identification. Pattern Anal. Mach. Intell..

[30] Luna C.A., Jiménez J.A., Pizarro D., Losada C., Rodriguez J.M. (2010). GPCA vs. PCA in recognition and 3-D localization of ultrasound reflectors. Sensors.

[31] Fernandez-Rincon A., Fuentes-Jimenez D., Losada-Gutierrez C., Marron-Romera M., Luna C.A., Macias-Guarasa J., Mazo M. Robust people detection and tracking from an overhead Time-of-Flight camera. Proceedings of the 12th International Joint Conference on Computer Vision, Imaging and Computer Graphics Theory and Applications.

[32] Macias-Guarasa J., Losada-Gutierrez C., Fuentes-Jimenez D., Garcia-Jimenez R., Luna C.A., Fernandez-Rincon A., Mazo M. (2016). GEINTRA Overhead ToF People Detection (GOTPD1) Database. http://www.geintra-uah.org/datasets/gotpd1.

[33] Khoshelham K., Elberink S.O. (2012). Accuracy and resolution of kinect depth data for indoor mapping applications. Sensors.

[34] Sahani M., Nanda C., Sahu A.K., Pattnaik B. Web-based online embedded door access control and home security system based on face recognition. Proceedings of the 2015 International Conference on Circuits, Power and Computing Technologies [ICCPCT].

